# *QuickStats:* Suicide Rates*^^,†^^ for Teens Aged 15–19 Years, by Sex — United States, 1975–2015

**DOI:** 10.15585/mmwr.mm6630a6

**Published:** 2017-08-04

**Figure:**
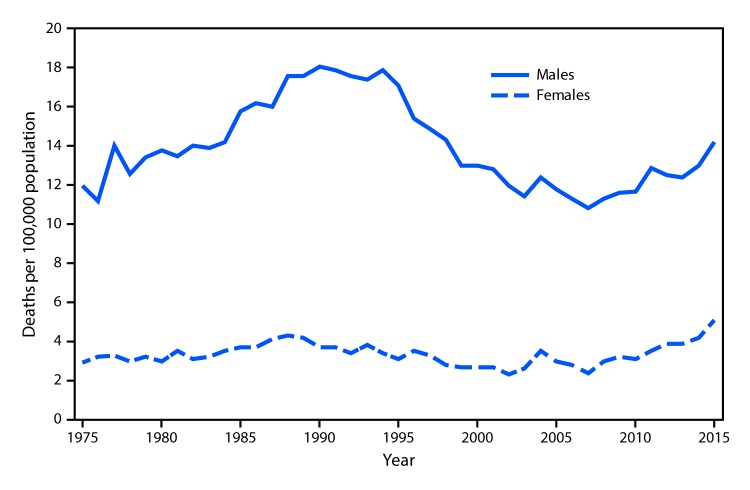
The suicide rate for males aged 15–19 years increased from 12.0 to 18.1 per 100,000 population from 1975 to 1990, declined to 10.8 by 2007, and then increased 31% to 14.2 by 2015. The rate in 2015 for males was still lower than the peak rates in the mid- 1980s to mid-1990s. Rates for females aged 15–19 were lower than for males aged 15–19 but followed a similar pattern during 1975–2007 (increasing from 2.9 to 3.7 from 1975 to 1990, followed by a decline from 1990 to 2007). The rates for females then doubled from 2007 to 2015 (from 2.4 to 5.1). The rate in 2015 was the highest for females for the 1975–2015 period.

**Source:** CDC. National Vital Statistics System, mortality data. https://www.cdc.gov/nchs/nvss/deaths.htm.

For more information on this topic, CDC recommends the following link: https://www.cdc.gov/violenceprevention/suicide/index.html

